# SNRAware: Improved Deep Learning MRI Denoising with Signal-to-Noise Ratio Unit Training and G-Factor Map Augmentation

**DOI:** 10.1148/ryai.250227

**Published:** 2025-10-22

**Authors:** Hui Xue, Sarah M. Hooper, Iain Pierce, Rhodri H. Davies, John Stairs, Joseph Naegele, Adrienne E. Campbell-Washburn, Charlotte Manisty, James C. Moon, Thomas A. Treibel, Michael S. Hansen, Peter Kellman

**Affiliations:** ^1^Health Futures, Microsoft Research, 14820 NE 36th St, Bldg 99, Rm 4941, Redmond, WA 98052; ^2^National Heart, Lung, and Blood Institute, National Institutes of Health, Bethesda, Md; ^3^Institute of Cardiovascular Science, University College London, London, UK; ^4^Barts Heart Centre, Barts Health NHS Trust, London, UK

**Keywords:** MRI, Deep Learning, MRI Denoising

## Abstract

**Purpose:**

To develop and evaluate a deep learning–based MRI denoising method using quantitative noise distribution information obtained during image reconstruction to improve model performance and generalization.

**Materials and Methods:**

This retrospective study included a training set of 2 885 236 images from 96 605 cardiac cine series acquired with 3-T MRI scanners from January 2018 to December 2020. Of these data, 95% were used for training, and 5% were used for validation. The hold-out test set included 3000 cine series, acquired in the same period. Fourteen model architectures were evaluated by instantiating each of the two backbone types with seven transformer and convolution block types. The proposed SNRAware training scheme leveraged MRI reconstruction knowledge to enhance denoising by simulating diverse synthetic datasets and providing quantitative noise distribution information. Internal testing measured performance using peak signal-to-noise ratio and structural similarity index measure, whereas external tests conducted with 1.5-T real-time cardiac cine, first-pass cardiac perfusion, brain, and spine MRI assessed generalization across various sequences, contrast agents, anatomies, and field strengths.

**Results:**

SNRAware improved performance on internal tests conducted on a hold-out dataset of 3000 cine series. Models trained without reconstruction knowledge achieved the worst performance metrics. Improvement was architecture agnostic for both convolution and transformer models. However, transformer models outperformed their convolutional counterparts. Additionally, three-dimensional input tensors showed improved performance over two-dimensional images. The best-performing model from the internal testing generalized well to external samples, delivering 6.5 and 2.9 times contrast-to-noise ratio improvement for real-time cine and perfusion imaging, respectively. The model trained using only cardiac cine data generalized well to three-dimensional T1-weighted magnetization-prepared rapid gradient-echo brain and T2-weighted turbo spin-echo spine MRI acquisitions.

**Conclusion:**

The SNRAware training scheme leveraged data obtained during the image reconstruction process for deep learning–based MRI denoising training, resulting in improved performance and good generalization.

**Keywords:** MRI, Deep Learning, MRI Denoising

*Supplemental material is available for this article.*

© The Author(s) 2025. Published by the Radiological Society of North America under a CC BY 4.0 license.

## Graphical abstract

**Figure:**
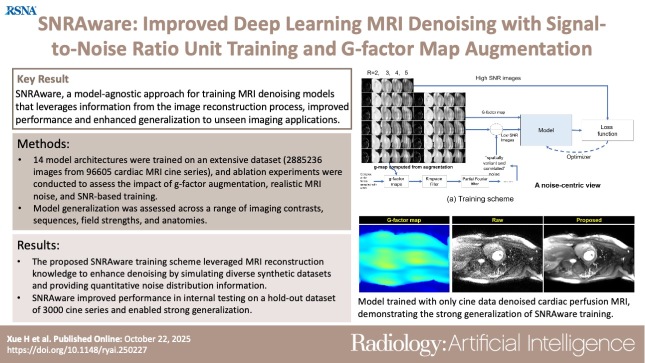

**Summary**

SNRAware, a model-agnostic approach for training MRI denoising models that leverages information from the image reconstruction process, improved performance and enhanced generalization to unseen imaging applications.

**Key Points**

■ In this retrospective study including 3000 cine series, the integration of quantitative noise-distribution information from signal-to-noise ratio unit reconstruction and geometry factor augmentation improved MRI denoising performance.
■ Two backbone types, HRnet and U-Net, were instantiated with seven transformer and convolutional block types to evaluate 14 architectures.
■ Models were trained on a dataset of 96 605 cine series and validated extensively on internal and external data, showing that the proposed method improved performance and generalization.

## Introduction

Deep neural networks outperform conventional filtering methods in restoring low signal-to-noise ratio (SNR) MRI acquisitions (1), benefiting applications such as low-field MRI (2,3), diffusion imaging (4), highly accelerated parallel imaging (5), and dynamic cardiac imaging (6). Deep learning–based MRI denoising further enhances diagnostic quality and clinical value.

Deep learning–based denoising can be performed using supervised or self-guided training. Supervised learning requires noisy-clean paired data, which can be difficult to acquire, especially for inherently low-SNR applications. To overcome this limitation, self-guided training (7–10) methods that use only noisy images have been proposed. These methods include Noise2Noise (8), which learns from paired noisy images, and Noise2Void (9) and Noise2Fast (10), which require only one noisy image and learn to predict blind-spot pixels from surrounding pixels or small patches from neighboring patches. These methods are slow at inference and underperform compared with supervised learning (11). Therefore, recent advances have adopted diffusion-based generative training, such as denoising diffusion models (4) or score-based diffusion sampling (12), to further enhance image quality.

Existing training approaches often overlook potential performance gains from noise information and are typically trained on limited datasets that include only specific imaging protocols and contrast agents (4,8,10,12). The resulting models, therefore, may not be robustly transferrable to other applications, particularly those with intrinsically low SNR where the initial image quality is poor and denoising is more challenging. However, MRI noise distribution can be derived from the image reconstruction process, enhancing model performance and generalization.

To take advantage of noise distribution data, we proposed SNRAware, an MRI denoising training scheme that leverages noise prewhitening (13) with real geometry factor (g-factor) maps to create spatially varying noise; noise correlation augmentation to integrate operations such as k-space filters, phase oversampling, and image resizing; and SNR unit reconstruction (14) to ensure a unity noise level and aid model learning. We also proposed a g-factor augmentation method to compute g-factor maps for acceleration factors of 2 to 8, even when data for a given acceleration are unavailable. This method contrasts with prior work typically trained on normalized signal, directly integrating noise into training and eliminating the need for paired high- and low-SNR images.

We trained models on an extensive dataset and conducted ablations to assess the impact of g-factor augmentation, realistic MRI noise, and SNR-based training, evaluating 14 architectures for model-agnostic improvements. We also aimed to investigate whether noise-centric training enhanced generalization to unseen applications across a range of imaging contrast agents, sequences, field strengths, and anatomies.

## Materials and Methods

### Data Collection

This retrospective study utilized retrospectively gated cardiac cine MRI data from 3-T clinical scanners (MAGNETOM Prisma; Siemens Healthineers) with a balanced steady-state free precession sequence. Consecutive data were acquired with an acceleration factor of 2 across standard cardiac views (two chamber, three chamber, four chamber, and short-axis stack), with raw k-space signals saved for reconstruction between January 2018 and December 2020. This dataset has not been used in previous publications.

Data from the National Institutes of Health Cardiac MRI Raw Data Repository, hosted by the Intramural Research Program of the National Heart, Lung, and Blood Institute, were curated with the required ethical and/or secondary audit use approvals or guidelines permitting the retrospective analysis of anonymized data without requiring written informed consent for secondary usage for the purpose of technical development, protocol optimization, and/or quality control. The data were fully anonymized and used for training without exclusion. The training and test datasets are summarized in Table 1. The training set included 96 605 cine series (2 885 236 images) from 7590 patients, with 95% of the scans used for training and 5% for validation, and the internal test set included 3000 cine series with no overlap.

**Table 1:** Imaging and Demographic Characteristics for Training and Test Datasets

Category	Imaging Application	Anatomy	Typical Sequence Parameters	Field Strength	No. of Samples and Data Format
Training and internal testing	Retro-gated cine	Heart	Data acquisition with breath holding FOV: 360 × 270 mm^2^ Acquired matrix size: 256 × 144 Echo time: 1.28 msec Bandwidth: 977 Hz/pixel Readout: SSFP RF flip angle: 50° Echo spacing: 2.97 msec Output phases: 30 Acceleration: R = 2	3 T	Training: *n* = 7590 patients; 96 605 cine series; 2 885 236 images, 61% male patients; mean age, 54 years Testing: *n* = 231 patients, 3000 cine series; 89 899 images 2D+T time series Input tensor: [B, 3, T, H, W]
Testing, external	Real-time cine	Heart	Data acquisition with single-shot free breathing FOV: 360 × 270 mm^2^ Acquired matrix size: 192 × 110 Echo time: 0.98 msec Echo spacing: 2.27 msec Bandwidth: 1100 Hz/pixel Readout: bSSFP RF flip angle: 50° Imaging duration: 39 msec Acceleration: R = 5	1.5 T	*n* = 10 patients; one section per patient; 8 male patients; mean age, 52 years 2D+T time series Input tensor: [B, 3, T, H, W]
Testing, external	Perfusion	Heart	Data acquisition with single-shot free-breathing Contrast material injection and dynamic contrast changes Adenosine stress FOV: 360 × 270 mm^2^ Acquired matrix size: 256 × 108 Echo time: 1.17 msec Single-shot repetition time: 80 msec Bandwidth: 850 Hz/pixel Readout: bSSFP RF flip angle: 50° Acceleration: R = 4	1.5 T	*n* = 5 patients; each had a stress and a rest scan; 3 sections per scan with 60 heart beats; 2 male patients; mean age, 43 years 2D+T time series Input tensor: [B, 3, T, H, W]
Testing, external	Neuro	Brain	T1-weighted MPRAGE sequence FOV: 250 × 250 mm^2^ Acquired matrix size: 256 × 256 Echo time: 7.2 msec Bandwidth: 250 Hz/pixel Readout: turbo spin echo Echo spacing: 3.58 msec Inversion time: 200 msec Acceleration: R = 2 × 2	1.5 T	*n* = 1 male; 45 years old Three-dimensional imaging Input tensor: [B, 3, D, H, W]
Testing, external	Spine	Spine	T2-weighted turbo spin-echo sequence FOV: 340 × 340 mm^2^ Acquired matrix size: 448 × 448 Echo time: 89 msec Repetition time: 3000 msec Bandwidth: 260 Hz/pixel Readout: turbo spin echo Acceleration: R = 2	1.5 T	*n* = 1 male; 45 years old 2D imaging for 15 sections Input tensor: [B, 3, SLC, H, W]

Note.—bSSFP = balanced steady-state free precession, FOV = field of view, MPRAGE = magnetization-prepared rapid gradient echo, RF = radiofrequency, 2D = two-dimensional.

Four external tests were conducted to evaluate generalization: *(a) *10 real-time cine sections with balanced steady-state free precession contrast but different sequence parameters, *(b)* five free-breathing first-pass perfusion scans for dynamic contrast changes, *(c)* a three-dimensional (3D) T1-weighted magnetization-prepared rapid gradient-echo (MPRAGE) brain scan (acceleration factor of 2 × 2), and *(d) *a high-resolution (0.76 mm^2^) two-dimensional (2D) T2-weighted turbo spin-echo multisection spine scan (acceleration factor of 2), all acquired at 1.5 T (MAGNETOM Aera; Siemens Healthineers). For 2D + T cases such as the cine series, the third dimension is time; while for 3D scans like the T1-weighted MPRAGE acquisitions of the brain, it is the second encoding dimension or depth; and for spine scans with multiple 2D sections, it is the section dimension. Phantom scans were also acquired at 1.5 T with acceleration factors of 2 and 4 using standard fast low-angle shot readouts.

### Training Method

SNRAware was aimed toward improving MRI denoising by generating low-SNR data and providing noise distribution data to the network. The data generation process is shown in Figure 1, while Figure 2 outlines the training scheme and model design.

**Figure 1:**
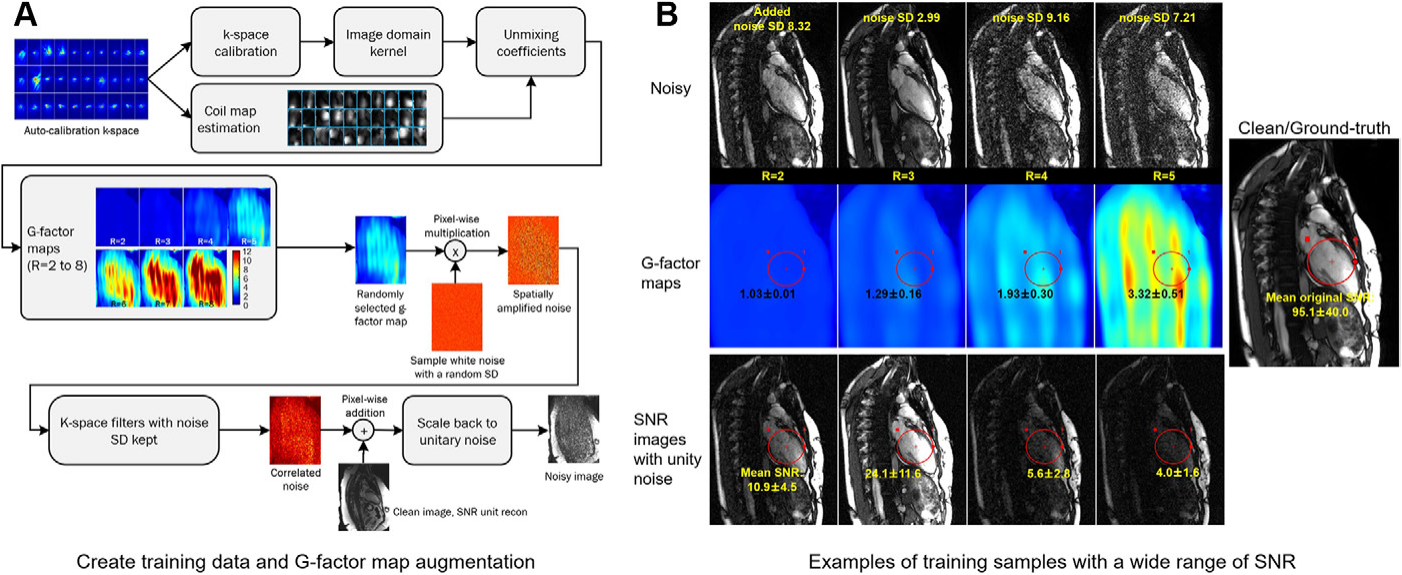
Training sample creation. **(A)** The training samples are paired clean- and noisy-image series. The noisy images are created by computing the geometry (g)-factor maps and using them to generate noise images. G-factor maps were computed from the autocalibration k-space. Generalized autocalibrating partial parallel acquisition (GRAPPA) calibration was computed for accelerations factors of 2 to 8. The GRAPPA k-space kernel was converted to image domain kernel, and unmixing coefficients were computed by combining image domain kernels and coil maps. For data augmentation, a g-factor map is randomly selected and pixel-wise multiplied to the white noise. The resulting spatially varying noise further goes through k-space filtering steps to introduce correlation. The final noise is added to the clean image and scaled to be unitary, as the noisy sample for training. **(B)** Four noisy samples are created from a clean cine series. The original signal-to-noise ratio (SNR) is 95.1 in the region of interest. By randomly selecting a g-factor map and changing the starting noise level, a wide range of SNRs can be produced. The SNR images are computed by dividing the images by the g-factor map.

**Figure 2:**
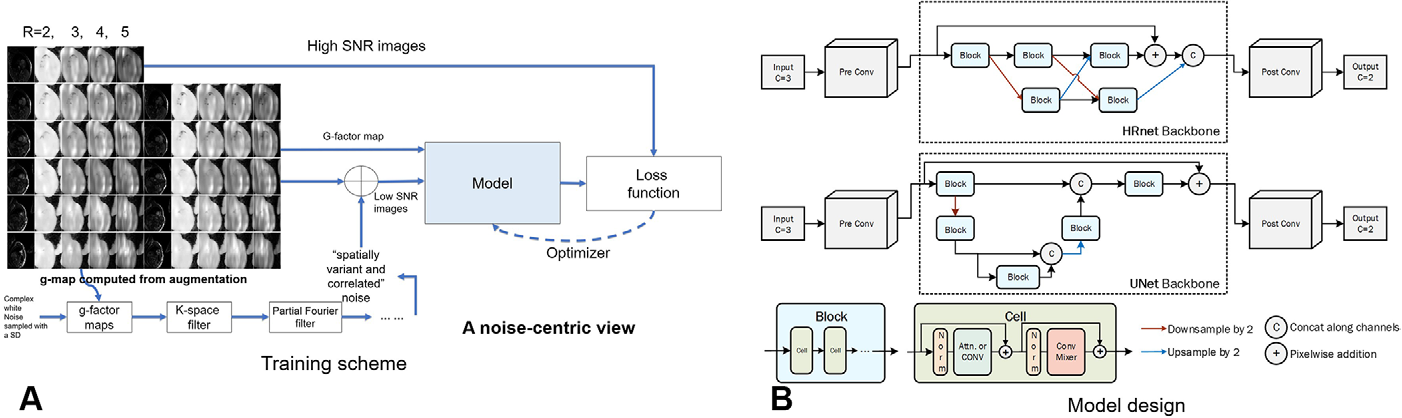
Overview of training scheme and model design. **(A)** Reconstructed cine images are augmented with spatially varying and correlated noise to create noisy samples. The corresponding geometry (g)-factor maps are concatenated to the images and used as input into the model. The model predicts high signal-to-noise ratio (SNR) images. **(B)** All models consist of a preconv layer as the shallow feature extractor, a backbone, and the output convolution. To simplify the evaluation on different models, a cell-block backbone design is proposed for the backbone. Two backbone architectures tested here are HRnet and U-Net. Both backbones process tensors through blocks which are connected by the downsample/upsample operation. Every block consists of three to six cells. Every cell follows the standard design, including normalization, attention or convolution layers, and skip connections. By changing the module in the cell, different transformers and convolution models are instantiated and tested.

**Training data generation with g-factor augmentation**

Training data were acquired with an acceleration factor of 2 undersampling to minimize g-factor–related noise amplification. To generalize higher accelerations (acceleration factor of 3 to 8), we proposed a g-factor–based data augmentation scheme to compute real g-factor maps at higher accelerations. With parallel imaging, noise scales with the g-factor due to the ill-posed inversion of the calibration matrix (15,16). However, this noise amplification varies from scan to scan. After reconstruction, the noise SD for pixel location *p* is *g*(*p*), where *g* increases dramatically at higher accelerations (Figs 2, 3).

**Figure 3:**
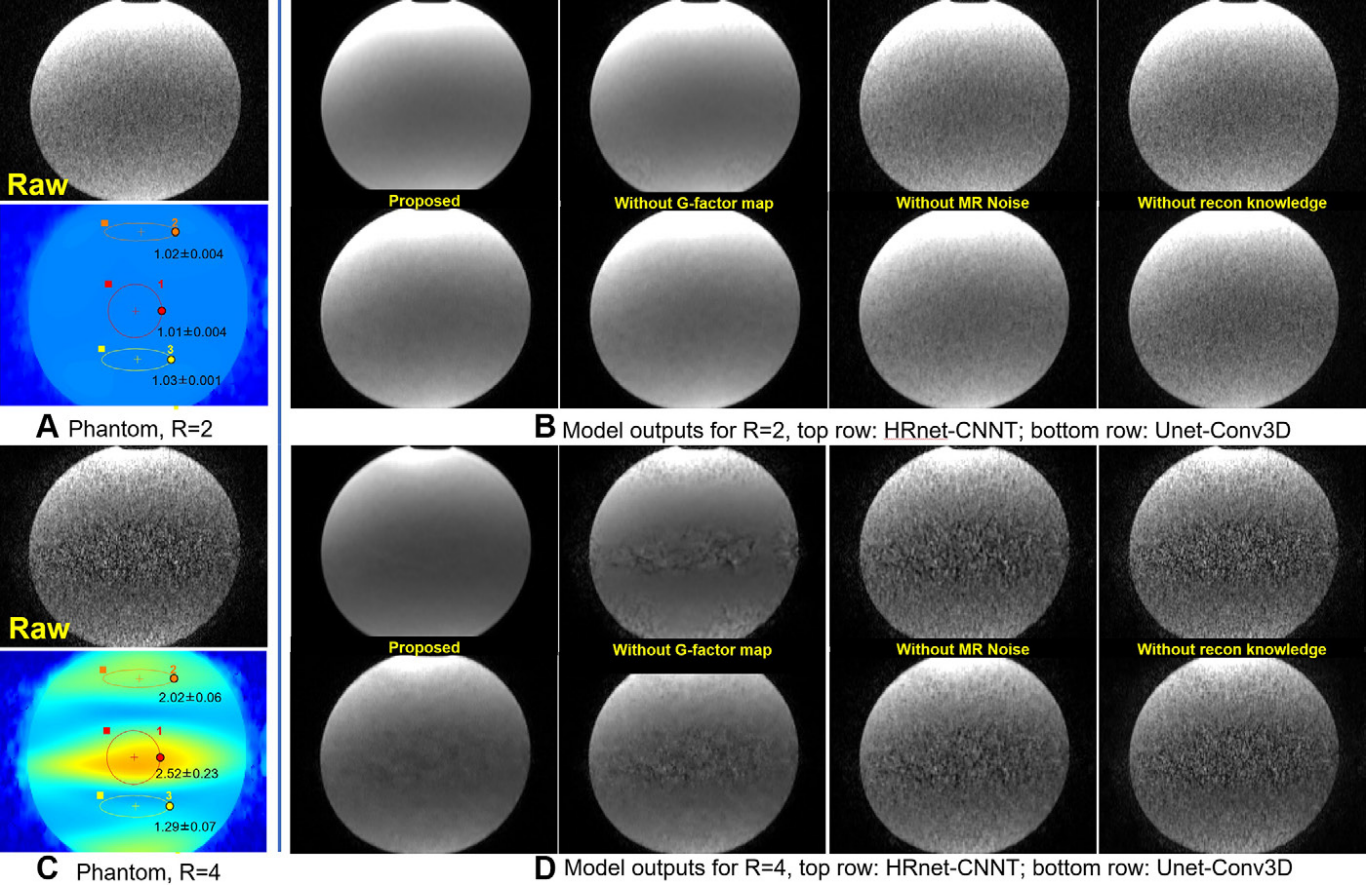
Phantom test results. **(A)** The raw images with acceleration factors of 2 and geometry (g)-factor map show a very minor noise amplification. **(B)** Images show model outputs with one transformer and one convolution architecture for proposed training, compared with ablation tests. **(C)** The images and g-factor map for acquisitions with acceleration factors of 4 show a lower signal-to-noise ratio and spatial noise amplification. The g-factor is higher at 2.52 at the center of field of view. The proposed method removes noise amplification. Training without g-factor map results in less efficient noise removal. For both accelerations, transformer models outperform convolution. Training without MR noise distribution further degrades the performance. CNNT = convolutional neural network transformer.

MRI systems acquire raw signals and store them in k-space, which represents the Fourier transform of the image intensities. To accelerate data acquisition, only a portion of the k-space data are collected. For instance, acquiring half of the data yields an acceleration factor of 2. Parallel imaging techniques are then used to estimate the missing k-space data to reconstruct the full image. This process, however, amplifies the noise present in the acquired signals, leading to reconstructed images with reduced SNR. G-factor maps computed during parallel imaging reconstruction quantify noise amplification, with each pixel value indicating exactly how much the noise has been amplified. Assuming SNR unit scaling has been applied and the original images have unitary noise (noise SD of 1.0), the g-factor directly reflects the postreconstruction noise SD. Of note, this noise amplification varies spatially among pixels due to the geometry and coupling of the receiver coils, the human body, and the specifics of the reconstruction algorithm. Therefore, the g-factor map provides crucial information about where noise is present and to what extent (Fig 1).

The SNRAware training theme (Fig 1A) computed g-factor maps from 2D generalized autocalibrating partial parallel acquisition coefficients derived from autocalibrated or fully sampled k-space lines. These coefficients were converted into image domain unmixing coefficients (17,18), and g-factor maps were obtained as the sum of their squares. Although the original scans used an acceleration factor of 2, g-factor maps for other accelerations were computed from corresponding unmixing coefficients. A randomly selected g-factor map was used to amplify white complex noise via pointwise multiplication during training.

Reconstruction steps such as k-space filtering and zero-filling resizing introduce spatial noise correlation to influence spatial noise distribution. To mimic these effects, we developed a training data augmentation process that varies noise correlation based on common reconstruction techniques by sampling white noise with a randomly selected ∑ (ranging from 0 to 32.0) from each training image. This noise was amplified by a g-factor map and then modified using a k-space filter (Gaussian filter: ∑ of 0.8, 1.0, 1.5, 2.0, or 2.25), after which a partial Fourier filter was applied with a probability of 0.5 (tapered Hanning filter [19]; partial Fourier sampling ratio of 1.0, 0.85, 0.7, 0.65, or 0.55). Reduced resolution was mimicked by masking out high-frequency samples (ratio: 1.0, 0.85, 0.7, 0.65, or 0.55). Each operation was independently applied to readout and phase-encoding directions.

By randomly selecting starting noise ∑, acceleration, and k-space filters, the augmentation procedure produced a wide range of spatially varying noise that closely resembles what would be observed with clinical imaging. Figure 1B shows noisy samples generated with different SNR levels (Movie 1).

**Movie 1:** The movies correspond to the example in Figure 1B. The reference standard clean image is the single one on the left. The first row are the noisy samples. The second row are the SNR images.

**Providing noise distribution information to the network**

We provided information about the noise distribution to the network to aid in the denoising task. Additionally, we used SNR unit (14) reconstruction for all training and test data to carefully scale the noise SD to unity and maintain this scaling through the reconstruction process. We aimed to determine if this method would aid the denoising model by reducing the variation in noise distributions which the model must learn. To perform SNR unit reconstruction, noise-only data were acquired before every scan (14). The noise readouts were used to compute the covariance matrix and perform noise prewhitening on the imaging readouts (17,20), and the noise SD was scaled to 1.0 by compensating for the equivalent noise bandwidth for every receiver coil or channel. The imaging data with unity noise went through fast-Fourier transform, parallel imaging generalized autocalibrating partial parallel acquisition reconstruction (16), and coil combination to produce the final complex images, with noise scaling kept constant throughout (14). The complex images were finally resized to the target matrix size with zero filling.

We utilized similar techniques to keep the noise SD constant when generating synthetic noise for our training data. Given a high-SNR image, *I*, which was reconstructed while maintaining unit noise variance scaling through all steps except parallel imaging unmixing, and a corresponding native g-factor, *g*, the corresponding SNR unit image is *S* = *I/g*. Generated correlated noise *n*, with a selected variance of σ^2^ and augmented as described above, was added to the SNR unit image to create a noisy sample: *S_n_* = (*S* + *n* × *g_aug_*)/√σ^2^ + 1, where *g_aug_* is a g-factor map computed by the aforementioned g-factor map augmentation. The ratio 1/√*σ*^2^ + 1 accounts for original unity plus added noise and returns the image scaling to unit noise variance excluding the noise amplification introduced by parallel imaging unmixing. Every training pair consisted of a clean sample, *S*, and noise augmented sample, *S_n_*. The clean image had unity noise, whereas the noisy image had spatial varying noise multiplied by* g_aug_*.

The process aforementioned is illustrated in Figure 2A. In addition to providing the network images with unity noise, we also provided the g-factor map to the network stacked along the channel dimension. This directly provided the network with information about the spatial amplification of noise in the image.

Sample code and detailed explanations can be found in previous publications and tutorials (13,17,18,20) for the noise prewhitening, SNR unit scaling, and computation of pixel-wise g-factor maps from parallel imaging calibration. Gadgetron framework *(https://github.com/gadgetron/gadgetron)* provided an open-source high-performance implementation.

### Model and Training

The inputs for all models were five-dimensional tensors—batch; channel; time, section, or depth; height; and width—providing the flexibility to support different imaging formats.

We evaluated 14 model architectures based on two adapted backbone types: HRnet (21) and U-Net (22) (Fig 2B). The transformer layers were inspired by the Swin (23), ViT (24), and more recent convolutional neural network transformer (CNNT) (11) models, where input tensors are split into patches across time, section, or depth; height; and width, and attention was computed over patches. Detailed backbone configurations can be found in Appendix S1.

The loss was the sum of Charbonnier loss (25), MRI perpendicular loss (26) designed to match complex values, visual geometry group perceptual loss on magnitude (27), and gradient loss, computed as the L1 difference of intensity gradient between reference standard and predicted tensors.

The dataset was split into 95% for training and 5% for validation. The model was trained with the Sophia (28) optimizer with the one-cycle learning rate scheduler (29) and cosine annealing. All models were implemented using PyTorch (30).

More information for model training is available in Appendix S2.

### Statistical Analysis

**Internal test**

Low-SNR images were generated for the test dataset of 3000 series using the aforementioned process. The resulting data were fed into the trained model, and peak SNR ($10\times log_{10}(\frac{MAX^{2}}{MSE});$ *MSE* = mean square difference, *MAX* = maximal value of image pixels) and structural similarity index measure (SSIM) (31) were computed for model outputs against the reference standard data. Since the image signals were floating values and noise level was unity, *MAX* was set to 2048.0, as SNR above this threshold was highly unlikely.

Peak SNR and SSIM were reported for 14 tested models (two backbone types; layer types: CNNT, CNNT-large, Swin3D, ViT3D, ViT2D, Conv3D, Conv2D). Next, the following ablations were performed: *(a)* without g-factor map, in which models were trained without the g-factor map as an extra input channel and inference did not consider the g-factor map; *(b) *without MRI noise, in which training included the g-factor map, but the generated noise was not transformed by filters and, instead, uncorrelated white noise was added to the high-SNR images, and *(c) *with magnitude training without imaging knowledge, in which, as simulation of reconstruction information was not available, training was conducted on magnitude images (as found in Digital Imaging and Communications in Medicine images), and g-factor maps were excluded while MRI acquisitions with realistic noise and magnitude images were used with a channel dimension size of one.

**External tests**

High-SNR reference-standard images were not available for the imaging data acquired with higher acceleration. Therefore, SNR gain was estimated using the Monte-Carlo simulation method (14) by repeatedly adding a fixed amount of noise to the input data for 64 times. The noise level in the model outputs was measured by computing the SD across repetitions, and the SNR increase was measured as the reduction of noise SD. Regions of interests were drawn in the myocardium and blood pool, from which SNR and the contrast-to-noise ratio (CNR; as $\frac{2\times \left(Signal_{blood}-Signal_{myo}\right)}{\left(noise_{blood}+noise_{myo}\right)}$) were measured.

A paired *t* test was performed, and a *P* value < .05 was considered statistically significant. Matlab R2022a (MathWorks) was used for statistical computing.

## Results

### Phantom

Figure 3 presents phantom test results with g-factor maps shown for scans with acceleration factors of 2 and 4. For an acceleration factor of 4, noise was elevated in the center of the water phantom. Denoising with HRnet-CNNT and U-Net-Conv3D improved performance, with the highest SNR achieved when reconstruction information was incorporated into model training, and the poorest results observed when reconstruction knowledge was excluded. The transformer-based CNNT model outperformed Conv3D.

### Internal Tests

Models trained with SNRAware performed the best, and removing g-factor or realistic noise knowledge degraded performance. Table 2 summarizes the internal tests and ablation studies. Detailed results are given in Appendix S3.

**Table 2:** Results of Internal Tests for Two Backbone Types

Backbone Type	No. of Parameters	Structural Similarity Index				Peak Signal-to-Noise Ratio			
		Proposed	Without G-Factor	Without MR Noise	Without Recon Knowledge	Proposed	Without G-Factor	Without MR Noise	Without Recon Knowledge
HRnet									
CNNT-large	54 678 306	0.70	0.56	0.37	0.38	54.90	48.14	41.31	40.48
CNNT	27 485 139	0.68	0.58	0.38	0.38	54.14	49.19	41.51	40.38
Swin3D	54 664 836	0.68	0.59	0.40	0.40	53.78	49.54	42.53	41.23
ViT3D	27 478 404	0.63	0.60	0.55	0.48	51.75	49.89	47.91	44.00
Conv3D	22 815 891	0.60	0.57	0.57	0.46	50.74	49.06	48.73	41.52
ViT2D	17 746 308	0.48	NC	NC	NC	46.96	NC	NC	NC
Conv2D	20 382 867	0.47	NC	NC	NC	46.67	NC	NC	NC
U-Net									
CNNT-large	48 880 418	0.70	0.55	0.38	0.37	54.70	47.65	41.53	40.32
CNNT	25 226 195	0.67	0.57	0.38	0.38	54.09	48.93	41.51	40.49
Swin3D	49 309 316	0.63	0.48	0.51	0.40	51.59	45.23	46.47	41.22
ViT3D	25 661 828	0.62	0.60	0.58	0.48	51.25	50.49	49.31	44.25
Conv3D	18 787 475	0.62	0.59	0.49	0.44	51.79	50.02	46.15	40.96
ViT2D	15 487 364	0.47	NC	NC	NC	46.53	NC	NC	NC
Conv2D	16 206 995	0.50	NC	NC	NC	47.43	NC	NC	NC

Note.—*Proposed* refers to training with g-factor map augmentation and realistic noise with the signal-noise-ratio unit scaling. *Without g-factor* refers to training and inference without inputting geometry-factor (g-factor) maps. *Without MR noise* refers to training with white noise, but still adding g-factor maps. *Without recon knowledge* refers to training without g-factor maps and adding white noise, and the signal-to-noise ratio unit scaling was not used. *P* < .001 for proposed method against three ablation tests for convolutional neural network transformer (CNNT)–large, CNNT, Swin3D, ViT3D and Conv3D. Conv = convolution, NC = not computed, SWIN = shifted window transformer, ViT = vision transformer.

### External Tests

Table 3 presents the SNR and CNR of external tests.

**Table 3:** Results for Real-Time Cine and Perfusion Generalization Tests

Real-Time Cine, R = 5						
Measurement			SNR		CNR	
ROI	Blood pool G-factor, 4.05 ± 1.04		Myocardium G-factor, 3.95 ± 0.80		Blood pool and myocardium	
Proposed	70.04 ± 11.70		20.40 ± 3.22		49.65 ± 9.80	
Raw	13.47 ± 4.85	*P* < .001	5.81 ± 2.20	*P* < .001	7.67 ± 2.98	*P* < .001
Without g-factor	22.19 ± 7.88	*P* < .001	7.39 ± 2.70	*P* < .001	14.80 ± 5.49	*P* < .001
Without MR noise	19.33 ± 6.78	*P* < .001	8.09 ± 2.91	*P* < .001	11.24 ± 4.18	*P* < .001
Without recon knowledge	18.43 ± 5.98	*P* < .001	7.64 ± 2.73	*P* < .001	10.79 ± 3.81	*P* < .001
Perfusion, R = 4						
ROI	Blood pool G-factor, 1.91 ± 0.52		Myocardium G-factor, 1.87 ± 0.44		Blood Pool and myocardium	
Proposed	74.05 ± 26.32		16.69 ± 5.86		57.37 ± 24.63	
Raw	24.54 ± 14.81	*P* < .001	4.51 ± 2.69	*P* < .001	20.03 ± 12.42	*P* < .001
Without g-factor	70.1 ± 31.543	*P* = .01	12.5 ± 6.25	*P* < .001	56.6 ± 27.28	*P* = .84
Without MR noise	59.05 ± 32.05	*P* < .001	9.96 ± 5.53	*P* < .001	49.09 ± 27.5	*P* = .001
Without recon knowledge	46.54 ± 23.58	*P* < .001	8.98 ± 5.21	*P* < .001	37.56 ± 19.43	*P* < .001

Note.—Unless otherwise noted, data are means ± SDs. Statistical significance tests were the proposed method against the raw and three ablation tests. The α level for significance is adjusted to be .05/4 = .0125 to count for four tests. A paired *t* test was performed between proposed method and three ablations. CNR = contrast-to-noise ratio, g-factor = geometry factor, SNR = signal-to-noise ratio, ROI = region of interest.

Figure 4 presents real-time cine results for acceleration factors of 4 with the balanced steady-state free precession readout acquired using a protocol different from training data. The proposed training approach produced the highest SNR with improvements (*P* < .001, Table 3). Differences between the model outputs and raw images showed that removed noise exhibited a pattern like the g-factor map. When the g-factor map was not used in training, amplified noise was removed to a lesser extent (Movie 2). Across 10 cases, the mean SNR increased by 5.2 times for the blood pool and 3.5 times for the myocardium, while CNR improved 6.5 times. Additional cine examples appear in Movie 3.

**Figure 4:**
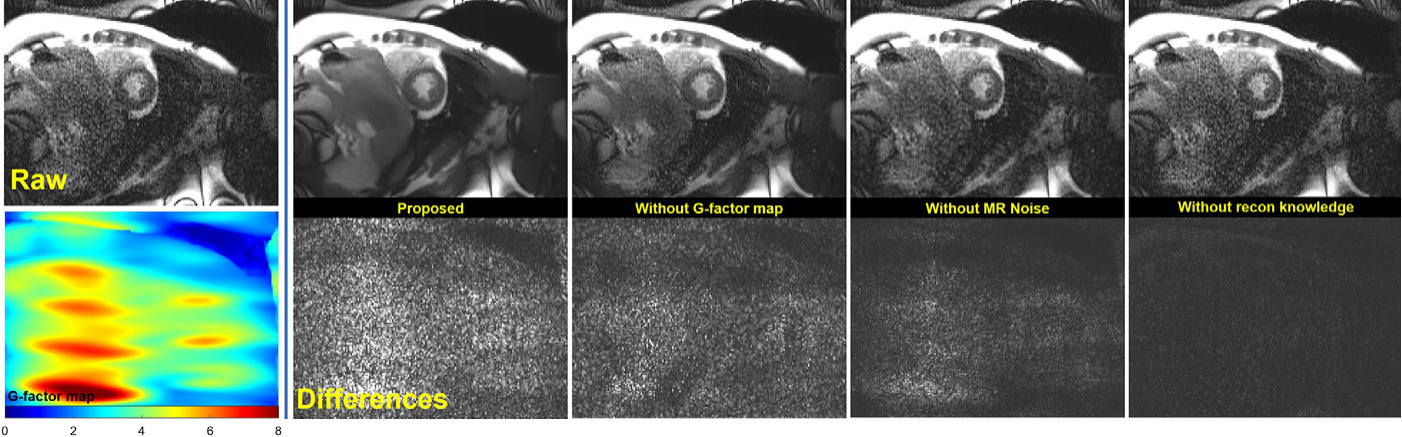
Images show real-time cine (acceleration, R = 5) results produced with the HRnet-convolutional neural network transformer (CNNT)–large model. The raw signal-to-noise ratio is lower with elevated spatial noise amplification due to acceleration. The proposed training method produced the best results.

**Movie 2:** Corresponding movies to Figure 4 are given here.

**Movie 3:** More R=5 real-time cine examples are given here. In all cases, proposed training noticeably improves performance. The leftover noise amplification is very visible without the g-factor map.

Figure 5 shows the results of perfusion imaging in which a contrast material bolus was passed through the heart. Perfusion dynamic contrast changes are different than the cine training data. The model trained with the addition of reconstruction knowledge successfully enhanced SNR across the field of view, whereas models lacking full imaging information performed worse. The proposed method increased the mean SNR by 3.0 times for the blood pool and 3.7 times for the myocardium, with a 2.9 times increase in CNR (Table 3). Movie 4 shows the corresponding videos.

**Figure 5:**
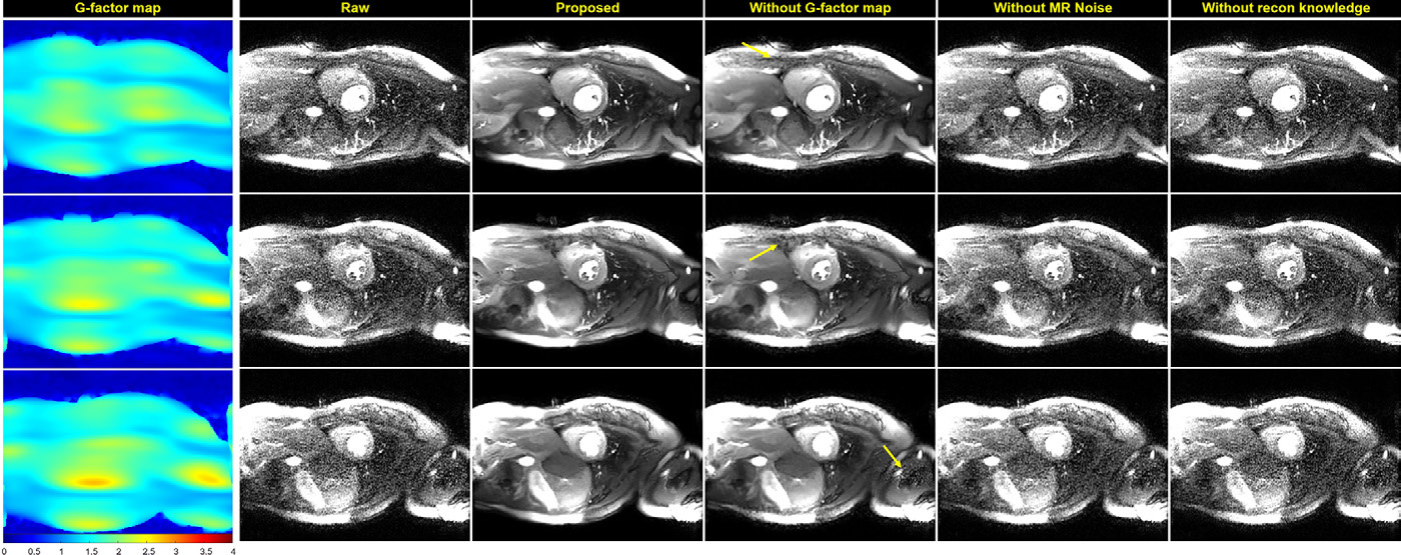
Images show results for accelerated (R = 4) myocardial perfusion imaging. The contrast passage creates dynamically varying contrast which was not seen in the training dataset. Moreover, the saturation preparation reduced the base signal-to-noise ratio. Despite the challenges, the model generalized well to perfusion imaging. Similarly to previous tests, noise reduction is more effective when knowledge about noise distribution is included in training.

**Movie 4:** Movies of perfusion denoising corresponding to Figure 5 are presented. Model generalized well to dynamic contrast and low base SNR.

Figure 6 demonstrates CNNT-large model generalization to unseen anatomies, including head and spine imaging. Despite differences in sequence, resolution, and contrast, the model improved SNR while preserving details. The white and gray matter contrast remained unchanged (Fig 6A), whereas a high-resolution T2-weighted turbo spin-echo spine scan (0.76 mm^2^) showed enhanced SNR in the vertebrae, disks, spinal cord, and cerebrospinal fluid (Fig 6B). Movies 5 and 6 show brain and spine results, respectively.

**Figure 6:**
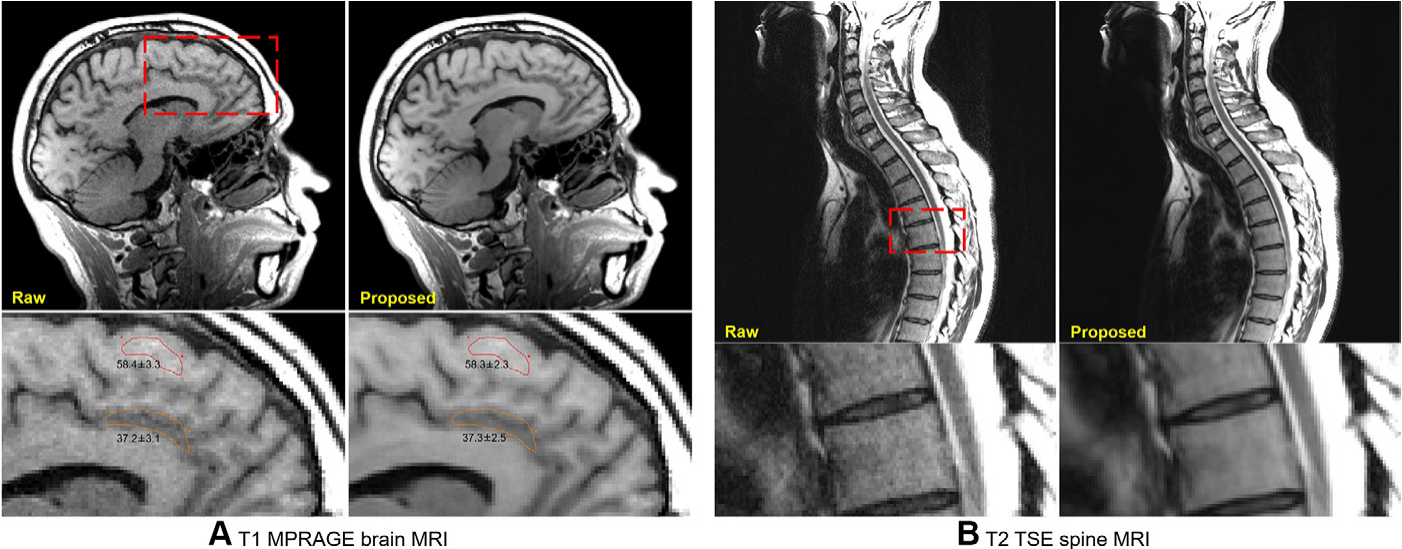
MR images show generalization tests for different anatomies. **(A)** A T1-weighted magnetization-prepared rapid gradient echo (MPRAGE) brain scan with an acceleration factor of 2 × 2 was acquired, reconstructed, and processed with trained model. The training dataset did not include any neuro data, yet the model generalized well, with noticeable signal-to-noise ratio improvement and preserved gray-white matter contrast. This test was to check whether models can generalize to new anatomies. Further evaluation is needed to test on more datasets with expert image reading. The gray and white matter differentiation appeared. **(B)** A T2-weighted turbo spin-echo (TSE) spine scan with an acceleration factor of 2 was processed with the trained model. Training data did not include spine scans and did not include the high spatial resolution (0.76 mm^2^) of this acquisition. The model generalized well to this application regardless.

**Movie 5:** Movie corresponds to Figure 6A for the T1 MPRAGE neuro test.

**Movie 6:** Movie corresponds to Figure 6B for the T2 TSE spine test.

## Discussion

We aimed to develop and evaluate a deep learning–based MRI denoising method using quantitative noise distribution information obtained during image reconstruction to improve model performance and generalization. To this end, this study introduced SNRAware, a new training scheme for MRI denoising that integrated knowledge from the image reconstruction process into deep learning. SNR unit reconstruction produced unit noise level images, simplifying the task of augmenting training data with realistic noise distributions. G-factor maps were added as model input data, providing quantitative information about spatially varying noise amplification. The proposed augmentation method subsequently computed real g-factor maps for acceleration factors of 2 to 8, requiring only a single acceleration factor for training data. MRI realistic noise was generated on the fly to lower the SNR of reference standard images, closely resembling the noise distribution on reconstructed images with higher acceleration factors and varying k-space filtering effects. This method eliminated the need for paired high- and low-SNR images for every acceleration factor and filter configuration, which is impractical for imaging with every acceleration or resolution. The training scheme was tested on 14 model architectures with two backbone types instantiated with transformer-based layers and convolution. Results of testing performed using phantoms, in vivo internal test data of 3000 cine series, and four external datasets showed that integrating reconstruction information into the training process consistently improved model performance. At higher acceleration factors, such as the acceleration factor of 5 real-time cine test, the proposed method effectively corrected g-factor noise amplification, whereas models trained without g-factor maps did not.

The clinical benefits of denoising models include improved SNR and imaging efficiency. The improved SNR enables faster acquisitions with higher acceleration, equating to cost and time savings which can shorten scan time, reduce sensitivity to motion, and can also be traded for increased spatiotemporal resolution. For example, thinner section thickness becomes more achievable, which reduces through-section dephasing and suppresses artifacts from implants. In a clinical study with inherently poor native SNR, such as low-field MRI, denoising models can play a key role in improving image quality for more accurate diagnoses (32).

The training method was model architecture–agnostic, as shown in the experiment where different models were trained with SNRAware. Leveraging quantitative noise distribution requires the availability of g-factor map information from the reconstruction process, a limitation of this method. The model inference in this method is fully automated and thus can be integrated in the clinical setting into the imaging workflow after reconstruction to process complex images with g-factor maps. The reproducibility of this method can be studied through the scan-rescan studies and expert image reading, enhanced with the quantitative measurement of SNR gain. Further analysis of noise characteristics can be found in Appendix S4. Furthermore, previous studies have proposed using g-factor maps in MRI denoising for CNN models (33,34). Appendix S5 gives an extended review on this topic.

This study does have some limitations. First, noise prewhitening is required for SNR unit scaling, necessitating noise calibration data, and although the Siemens scanners used in this study automatically acquire noise readouts, some curated raw datasets (such fastMRI data [36]) do not come with noise scans. Therefore, the application of this method to raw datasets that lack noise scans is limited. Second, the introduction of new processing steps that alter noise distribution augmentation would require an extension of the framework. With some generalization tests, therefore, more evaluation is needed for other contrast agents, resolutions, and anatomies. Third, global image quality metrics were used to measure improvement added by introducing imaging and reconstruction knowledge into the model training. Although this allows the comparison of different models, it is not a substitute for clinical evaluation, meaning that radiologists’ subjective assessments and diagnostic performance validation are still required to integrate models into the imaging workflow. Fourth, a single data source was used in our study; all data were acquired using Siemens MRI scanners at one site. Therefore, more evaluations are needed for a multicenter setup. Fifth, training data diversity was limited, as cardiac cine data were the only type of data used for training the network. Therefore, we assess how the addition of more diverse data for static image volumes improves model generalization over other anatomies. Finally, we conducted ablation tests with well-controlled training setups to demonstrate the added value of using quantitative noise information in denoising training. Although the improvements were agnostic to specific model architecture, we did not attempt end-to-end comparisons with other methods and systems, which would be crucial to the validation of models deployed into the clinical imaging workflow. In general, limitations such as a single data source, limited training data diversity and clinical validation, and noise prewhitening were acknowledged and are potential scopes for future studies.

In summary, this study used SSIM and peak SNR to compare different training setups and evaluate whether integrating noise information from the MRI reconstruction process would boost model performance. However, global image quality metrics were used to compare two models, rather than validating diagnostic performance. Previous studies (35) showed that SSIM correlated positively with the radiologists’ scores but tended to overlook local degradation, given that it is a global quality metric. This study focused on showing that the proposed training scheme would improve all model architectures. In future studies, therefore, dedicated radiologist evaluations are needed to validate model performance on a per-imaging-task basis. Models trained to recognize MRI noise distribution may generalize to unseen imaging applications, supported by experiments on perfusion imaging with dynamically changing contrast and brain and spine scans.

## Supplemental Files

Appendices S1-S5, Figures S1-S2

Conflicts of Interest
