# Supplementary material for: SNRAware: Improved Deep Learning MRI Denoising with Signal-to-Noise Ratio Unit Training and G-Factor Map Augmentation
Source: Radiol Artif Intell. 2025 Oct 22;7(6):e250227. doi: 10.1148/ryai.250227 (PMC12665503; doi:10.1148/ryai.250227)
Supplement: Supplementary file 1 [file ryai250227suppa1.pdf]

Supplemental materials undergo peer review but are not copyedited, and may include typographical errors.

©The Author(s) 2025. Published by the Radiological Society of North America under a CC BY 4.0 license.
10.1148/ryai.250227

Supplemental Appendices

Appendix S1. Information for deep learning models

As shown in Figure 2, the model consists of three components: pre-convolution layer, backbone and post-convolution layer. The input tensors are in the shape of [B, C, T/S/D/Z, H, W]. C is 3 for complex inputs (real, imagery and g-factor). Noise in the input images are scaled to 1.0×g-factor, as this setup is consistent with reconstruction outputs.

The pre-convolution layer is a shallow feature extractor (37). It is kept being minimal as a 2D convolution to uplift input channel C to 64, encouraging backbone to take on most heavy lifting and helping generalization. The post-convolution is another CONV layer, converting $C_{backbone}$ after the backbone to required output channels (2 for complex training and 1 for magnitude training).

Two well-known backbone architectures, HRnet and Unet, are implemented and tested in this study. Both architectures utilize the multi-resolution pyramid to balance model size, expressive power and computing cost. The building components include multiple Blocks, downsample and upsample layers, channel-wise concatenation, and skip connection. The HRnet maintains a longer pipeline on the original tensor size and Unet is smaller in size and less computing expensive.

Supplemental materials undergo peer review but are not copyedited, and may include typographical errors.

The input tensors are processed through every block, gaining more channels and reducing spatial resolution, which is explained by the backbone plots annotated with tensor sizes.

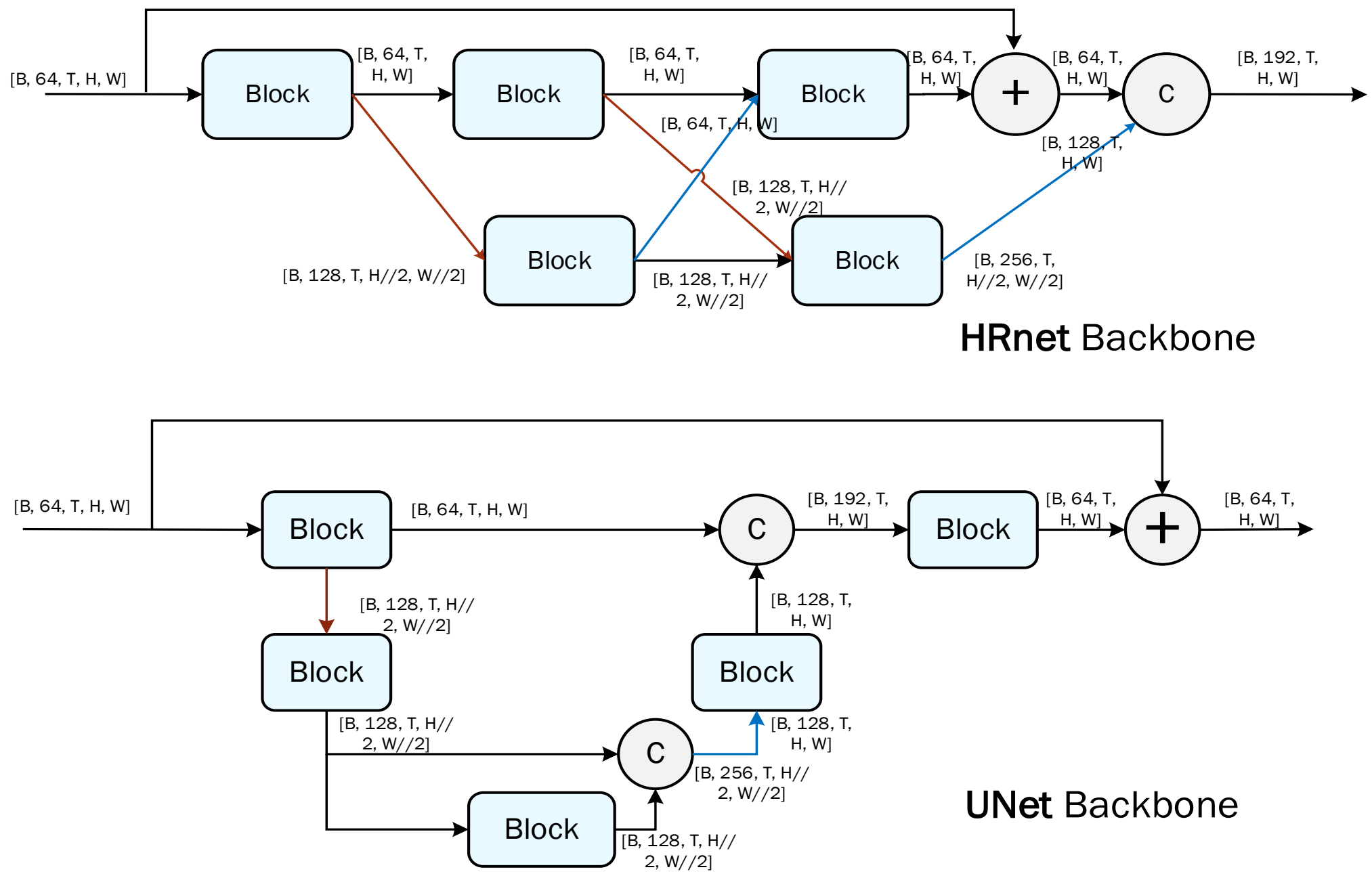

Figure 1. Annotated backbone architectures.

Downsampling was implemented with patch merging (23) followed by a convolution to format outputs to have the required number of channels. The upsampling was implemented with a linear interpolation followed by a CONV layer.

Backbones consist of several blocks. A block is a container of N cells. Every cell has a classical setup of two skip connections, layer norms and attention or convolution layers. By switching the attention methods (e.g. Swin3D, ViT3D or CNNT etc.), we can instantiate different models for experiments. A pure convolution model was implemented by replacing attention with convolution layers.

Every block in all models, except CNNT-large, has 3 cells. For CNNT-large, a block holds 6 blocks. By inserting more cells or more blocks, the model can be scaled up or down.

As used in other denoising training schemes, models were trained on image patches to encourage models to focus on noise distribution instead of image content. The patch size was [T/S/D/Z=16, H=64, W=64]. The window size in Swin3D and ViT3D was [16, 8 ,8], where every [2, 2, 2] neighborhood was processed as a token. The CNNT transformer method computed attention between all [H, W] frames without explicit neighborhood tokenization. All convolutions had the kernel size 3 and padding 1. We note that unlike the original Swin and ViT papers, we re-patch and un-patch the tensors before and after every operation, resulting

Supplemental materials undergo peer review but are not copyedited, and may include typographical errors.

in imaging tensors that can be processed by the normalization and convolutional mixer layers in every cell.

Appendix S2. Information for model training

The inputs for all models were 5D tensors—batch, channel, time or slice or depth, height, and width (B, C, T/S/D, H, W)—providing the flexibility to support different imaging formats. For instance, for the input cine series, the third dimension was time, whereas for a 3D brain scan it was depth or slice. The g-factor map was concatenated to real and imaginary parts of image tensors, so C was 3 for complex training, whereas if only the magnitude image was used, C was 2. The model outputs a tensor with the same shape with a channel dimension of 2 for complex and 1 for magnitude images, respectively.

We evaluated 14 model architectures based on two adapted backbone types: HRnet (21) and Unet (22) (Figure 2B), both of which use multi-resolution pyramids to balance computational complexity with the ability to recover small image features by maintaining a full resolution path. Each network consisted of multiple blocks containing several cells, with every cell including normalization, a computing layer, and a mixer. Different models were instantiated by configuring different computing layers, and both transformer and convolution layers were tested. The transformer layers were inspired by the Swin (23), ViT (24) and more recent CNNT (Convolutional neural network transformer) (11) models, where input tensors are split into patches across T/S/D, H, W and attention was computed over patches. For Swin, we split the input image into patches and applied attention over local and shifted windows; for ViT, attention was global among all patches; and the CNNT cells did not patch the image, instead applying attention in the T/S/D dimension. We also tested convolution layers ("Conv" blocks), which did not patch the image, instead applying standard convolution. All cells included three layers except for CNNT-large, with six layers. The ViT2D and Conv2D models were further trained through 2D patching and attention or 2D convolution, operating over height and width rather than across frames. These configurations enabled us to assess SNRAware over transformer, convolutional models, and 2D/3D models, as well as multiple backbone configurations (Supplemental Appendix S1).

The loss was the sum of Charbonnier loss (25), MRI perpendicular loss (26) designed to match complex values, VGG (Visual Geometry Group) perceptual loss on magnitude (27), and gradient loss, computed as the L1 difference of intensity gradient between reference standard and predicted tensors.

The dataset was split into 95% for training and 5% for validation. A fast second order optimizer, Sophia (28), was used with the one-cycle learning rate scheduler (29) and cosine annealing. The peak learning rate was 1e-5, betas were 0.9 and 0.999, and epsilon was 1e-8. The training lasted 80 epoch, and the final model was selected as the one giving the highest performance on the validation set. All models were implemented using PyTorch (30) and training was performed on a cluster of 128 AMD MI300X GPUs (Graphic Processing Unit), each with 192 GB RAM (Random Access Memory). Data distributed as used GPU cards to speed up training.

Supplemental materials undergo peer review but are not copyedited, and may include typographical errors.

Our training and model architecture design were generalized over various data dimensionalities by processing tensors in the shape of batch, channel, time/slice/frame, height and width as [B, C, T/S/D, H, W]. The models denoised each of these formats despite being trained on only 2D + T data, making it practical to combine different training data (e.g., 2D + T, 3D, and multi-slices) into one model training session, potentially improving model generalization.

Appendix S3. Internal and ablation results

Table 2 summarizes the internal test results. Ablation studies were conducted for all 3D and transformer models, but not for 2D models as they were much less competitive. The proposed scheme consistently yielded the best PSNR and SSIM. Removing g-factor or realistic MRI noise from model training degraded performance, with the poorest results observed when reconstruction knowledge was omitted.

Across the various architectures, CNNT-large performed better (as the highest scores in Table 2), with HRnet-CNNT-large achieving the highest PSNR (54.90) and SSIM (0.71). Comparing 3D and 2D versions (Hrnet backbone: ViT3D SSIM = 0.63 and PSNR = 51.75 vs. ViT2D SSIM = 0.48 and PSNR = 46.96, Conv3D SSIM = 0.60 and PSNR = 50.74 vs. Conv2D SSIM = 0.47 and PSNR = 46.67; Unet: ViT3D SSIM = 0.62 and PSNR = 51.25 vs. ViT2D SSIM = 0.47 and PSNR = 46.53, Conv3D SSIM = 0.62 and PSNR = 51.79 vs. Conv2D SSIM = 0.50 and PSNR = 47.43), 3D models showed superior performance, and the model with highest scores, Hrnet-CNNT-large, was used in the generalization tests. Table 3 presents the SNR and CNR results for cardiac generalization tests.

Appendix S4. More discussion about MR noise characteristics

MR noise follows the Gaussian distribution in real and imaginary components, after the noise pre-whitening [1]. The noise corrupts the signal as the additive component. The MR SNR will be biased higher if magnitude detection was applied. This bias decreases with more receiver channels and higher signal strength [2]. But in this study, the processing was in the complex domain, and we studied the noise distribution in the absence of signal as a training augmentation. As a result, the noise distribution was still Gaussian, and simulated noise can be added to the signal.

The uncorrelated white noise, however, will become correlated colored noise after MR reconstruction. The g-map noise amplification leads to spatially variant SNR, further deviating the noise distribution from the normal Gaussian.

Although it is tractable to analytically track the change of noise distribution, it is much easier to set up the deep learning training by sampling the white noise and passing them through the same processing steps and adding resulting noise to reduce image SNR. This is the approach used in this study, utilizing the additive nature of MR noise corruption.

Supplemental materials undergo peer review but are not copyedited, and may include typographical errors.

The results showed denoising model performance was improved if added noise was augmented with g-factor map and processed with the same filters as in the reconstruction. As the open-source Gadgetron MR reconstruction was used in this study, these requirements were precisely met in the model training, leading to noticeable boost in denoising performance.

To illustrate how the g-factor amplification and other steps alter the noise distribution, we sampled white noise and processed it with a real g-factor map and k-space filters. The noise power spectrum was plotted for the demonstration.

A noise was sampled for a 256×192 matrix. A R=5 g-factor map was sampled to amplify the noise. K-space filters were generated with filter width being 1.0 pixel, 1.5pixel and 2.5 pixel. Partial Fourier filters [3] were tapered Hanning with 6/8 sampling along the phase encoding (in this example, 192 is the phase encoding length) and a transition band of 12, 24 and 48 pixels. The power spectrum was computed by repeating the noise sampling 256 times and taking the average. The 1D profile of power spectrum was plotted here (along with the phase encoding direction at the k-space center) for visualization.

As shown in Figure S2, the g-map amplification and other processing steps changed the noise distribution and altered its appearance. Experiments showed model performance degraded if model was trained without realistic noise added. By randomly concatenating g-factor maps and filters, the training can see a wide range of combinations of colored noise, helping model differentiate signal from noise.

Supplemental materials undergo peer review but are not copyedited, and may include typographical errors.

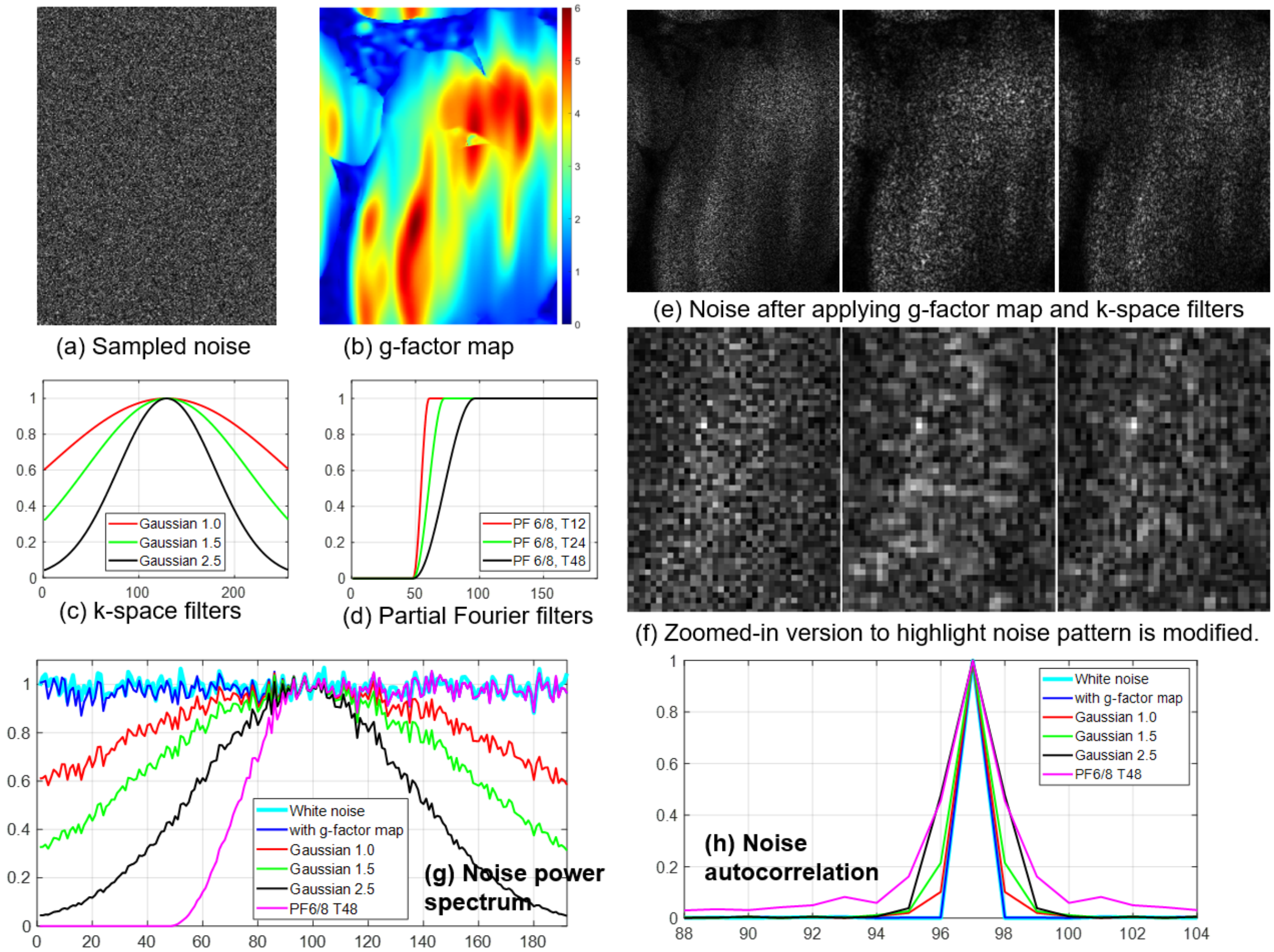

Figure S2. Demonstration of noise distribution after g-factor amplification and k-space filters. (a) The white noise was sampled to a 256x192 matrix. (b) A R=5 g-factor map was used in this demo. (c) A set of k-space filters are plotted for different strength. (d) The partial Fourier filters used in Gadgetron was on side Hanning with different transition bands. (e) Noise pattern after applying g-factor map and k-space filters. From left to right: only applying g-factor map; g-factor map and Gaussian 2.5 filter for both readout and phase encoding; g-factor map and Gaussian 2.5 filter for readout and PF 6/8 T48 filter for phase. (f) Zoomed-in version to visualize differences in noise pattern. (g) Power spectrum of noises. Note the flat white noise spectrum was altered by g-factor map and filters. Different combination of these processing steps will alter the noise differently. (h) Corresponding autocorrelation.

1. Gudbjartsson H, Patz S. The Rician distribution of noisy MRI data. Magn Reson Med. 1995;34(6):910–914. doi: 10.1002/mrm.1910340618.

2. Constantinides CD, Atalar E, McVeigh ER. Signal-to-noise measurements in magnitude images from NMR phased arrays. Magn Reson Med. 1997;38(5):852–857. doi: 10.1002/mrm.1910380524.

Supplemental materials undergo peer review but are not copyedited, and may include typographical errors.

3. Mcgibney G, Smith MR, Nichols ST, Crawley A. Quantitative Evaluation of Several Partial Fourier Reconstruction Algorithms Used in MRI. Magn Reson Med. 1993;51–59. https://doi.org/10.1002/mrm.1910300109.

Supplemental materials undergo peer review but are not copyedited, and may include typographical errors.

Appendix S5. Extended review for methods using g-factor maps

Previous studies have proposed using g-factor maps in MRI denoising. In a recent study (33), 23 T2 brain scans were collected and used to train a CNN (Convolutional Neural Network) model. G-factor maps were computed and multiplied with the MRI surface coil inhomogeneity maps, and the resulting "noise map" was used to scale the white noise to introduce spatial variance. The main difference when compared with SNRAware is that the previous study used g-factor maps for training data simulation with an unknown noise level; therefore, images were not scaled in the SNR units.

Another study (34) provided g-factor maps as input for training with low SNR. In that study a CNN model was trained with 2000 T2 brain scans and simulated noise. Internal testing was performed against other brain images; however, noise pre-whitening with actual noise readouts was not performed to get a fixed scaling level, rather the authors of that study estimated noise sigma using a wavelet method from the *k*-space data. We trained models on larger datasets for both transformers and convolution architecture, and the impact of noise amplification from the g-factor and noise correlation caused by raw filter and other steps were separately tested with more extensive out of distribution validations. Our study emphasized that a noise-centric view of denoising training can improve the generalization of trained models to unseen imaging applications.
